# Association of Breastfeeding Difficulties and Perceived Emotional Support with Retrospectively Reported Early-Postpartum Emotional Symptoms: A Cross-Sectional Study in Croatia

**DOI:** 10.3390/healthcare14213680

**Published:** 2026-10-30

**Authors:** Katija Sessa, Krešimir Dolić, Ajka Pribisalić, Mia Rogošić, Matea Dolić

**Affiliations:** 1 Faculty of Health Science, University of Split, 21000 Split, Croatia; katija.leas5@gmail.com (K.S.); kdolic@mefst.hr (K.D.); mia.rogosic@fzz.unist.hr (M.R.); 2 School of Medicine, University of Split, 21000 Split, Croatia; 3 University Hospital of Split, 21000 Split, Croatia

**Keywords:** breastfeeding difficulties, early-postpartum emotional symptoms, perceived emotional support, postpartum wellbeing, maternal mental health, midwifery

## Abstract

**Highlights:**

**What are the main findings?**

- Breastfeeding difficulties during the first 14 days postpartum were associated with higher early-postpartum emotional symptom scores in both unadjusted and covariate-adjusted analyses (adjusted B = 0.490, 95% CI 0.350–0.631, *p* < 0.001).
- Among women reporting breastfeeding difficulties, greater breastfeeding-related emotional burden was associated with higher emotional symptom scores, with the highest burden rating (rating 5 vs. 1) showing a statistically significant association in the adjusted model (B = 0.721, 95% CI 0.415–1.028, *p* < 0.001). Greater perceived emotional support was associated with lower symptom scores, whereas the breastfeeding-difficulty × perceived-emotional-support interaction was not statistically significant.

**What are the implications of the main findings?**

- Postpartum care may benefit from assessing breastfeeding difficulties together with maternal emotional wellbeing, particularly during the first two weeks after childbirth.
- Accessible professional and emotional support from partners, family members, community health nurse, midwives, and lactation consultants may help identify and support mothers experiencing greater early-postpartum distress.

**Abstract:**

Background: Breastfeeding difficulties and perceived support may be related to maternal emotional wellbeing, but their associations with early emotional symptoms require investigation. Objective: To examine associations between retrospectively reported breastfeeding difficulties, breastfeeding-related emotional burden, perceived emotional support, and early-postpartum emotional symptoms among mothers in Croatia. Methods: A cross-sectional online survey included 1378 women who had given birth within the preceding 12 months and reported their experiences during the first 14 days postpartum. Emotional symptoms were assessed using an investigator-developed, non-diagnostic five-item score (range 1–5). Analyses used nonparametric tests, Spearman correlations, and covariate-adjusted linear regression. Moderation by perceived emotional support was tested. Results: Participants reporting breastfeeding difficulties had higher symptom scores than those without difficulties (3.28 vs. 2.72; adjusted B = 0.490, 95% CI 0.350–0.631, *p* < 0.001). Among women reporting difficulties, emotional burden was positively correlated with symptom scores (Spearman’s ρ = 0.347; *p* < 0.001), and the burden rating (5 vs. 1) was associated with higher scores after adjustment (B = 0.721, 95% CI 0.415–1.028; *p* < 0.001). Perceived emotional support was negatively associated with symptom scores (B = −0.241, 95% CI −0.287 to −0.195; *p* < 0.001). The breastfeeding-difficulty × perceived-emotional-support interaction was not significant (B = −0.052, *p* = 0.381). Findings were consistent after excluding women reporting symptoms lasting longer than 14 days. Conclusions: Breastfeeding difficulties and greater breastfeeding-related emotional burden were associated with higher early-postpartum emotional symptoms, whereas greater perceived emotional support was associated with lower scores. No moderation by perceived emotional support was detected.

## 1. Introduction

The postpartum period is a dynamic phase of maternal adaptation that involves substantial physical, physiological, psychological, and social changes [1,2]. Breastfeeding is recommended because of its well-established benefits for both infants and mothers, and international guidance promotes exclusive breastfeeding during the first six months of life [2,3]. Population-level surveillance further shows that breastfeeding initiation, duration, and exclusivity remain important maternal and child health indicators [4]. At the same time, the establishment of breastfeeding occurs during a period of major maternal adjustment. Interventions aimed at strengthening postpartum social support [5], as well as research on the psychological dimensions of breastfeeding [6,7], indicate that feeding experiences and maternal emotional wellbeing are closely interconnected. Early breastfeeding may be complicated by nipple trauma, pain, breast engorgement, perceived insufficient milk supply, mastitis, and difficulties with infant latch [8,9,10]. These difficulties can be physically demanding and may also contribute to frustration, guilt, fear of failure, reduced confidence, and emotional burden, particularly when expectations regarding breastfeeding are high and professional support is perceived as insufficient. Emotional, informational, and practical support from personal and professional support networks may therefore be particularly relevant when mothers encounter early breastfeeding difficulties [5].

Maternity blues, also referred to as baby blues, is generally described as a mild and transient cluster of emotional symptoms arising in the first days after childbirth, including tearfulness, mood lability, anxiety, irritability, and emotional sensitivity [1,11]. A Croatian study reported maternity-blues symptoms in the immediate postpartum period and identified associations with selected psychosocial and obstetric factors [11]. Maternity blues should, however, be distinguished from postpartum depression and other clinically significant postpartum mental-health conditions. The broader literature on postpartum depression includes evidence on prevention [12], the relationship between maternal mood and breastfeeding [13], and established risk factors for postpartum depression [14]. Social support and breastfeeding self-efficacy are also relevant to postpartum adaptation [15], and a recent systematic review identified social support as an important coping resource across postpartum psychosocial outcomes [16]. A systematic review and meta-analysis of maternity blues reported substantial heterogeneity in reported occurrence across studies, reflecting differences in populations, timing, and measurement approaches [17]. Smaller studies have also reported associations between social or professional support and baby-blues-related outcomes [18], while a recent review emphasised the need to distinguish transient postpartum emotional changes from more persistent postpartum disorders [19]. Similar associations between family support and baby-blues-related outcomes have been reported in other postpartum samples [20].

Despite the recognized importance of breastfeeding support and maternal emotional health, the association between early breastfeeding difficulties, their perceived emotional burden, perceived emotional support, and early-postpartum emotional symptoms remains incompletely characterized, particularly during the first weeks after childbirth [18,19,20].

This study therefore examined retrospectively reported breastfeeding difficulties, emotional burden related to those difficulties, perceived emotional support, and early-postpartum emotional symptoms referring to the first 14 days after childbirth among women who had given birth within the previous year. Because the questionnaire was completed retrospectively and the emotional-symptom measure was investigator-developed and non-diagnostic, the study was designed to evaluate associations rather than to estimate the prevalence of maternity blues or establish causal relationships. Based on the literature described above, the following hypotheses were tested:

**H1.**  *Mothers who experienced breastfeeding difficulties would report higher early-postpartum emotional symptom scores than mothers without breastfeeding difficulties*.

**H2.**  *Among mothers reporting breastfeeding difficulties, greater emotional burden related to those difficulties would be associated with higher early-postpartum emotional symptom scores.*

**H3.**  *Greater perceived emotional support would be associated with lower early-postpartum emotional symptom scores.*

**H4.**  *Perceived emotional support would moderate the association between breastfeeding difficulties and early-postpartum emotional symptom scores.*

## 2. Materials and Methods

### 2.1. Study Design

This cross-sectional study used an anonymous online survey to assess retrospectively reported breastfeeding experiences, perceived emotional support, and emotional symptoms during the first 14 days after childbirth. Therefore, the evidence found in the article can only suggest associations between the analyzed variables and cannot establish temporal relationships or causation.

### 2.2. Participants and Recruitment

Eligible participants were mothers aged 18 years or older who had given birth within the previous year. Participants were recruited via convenience sampling through the Croatian Facebook groups in which mothers exchange experiences: Porod u Hrvatskoj–razmjena iskustva, Mame recenziraju, Trudnice & mame, Trudnice i mame sjeverne Hrvatske, Trudnice i mame: podrška i savjeti, Trudnice i mame–iskustva, savjeti, Mamine tajne anonimne i javne, Mame iz Solina, and Mame Splitsko-dalmatinske županije. The survey was additionally shared through private channels via the mobile applications Viber and WhatsApp. Data were collected between 22 April and 8 May 2026. A total of 1378 participants were included in the analysis. No a priori sample-size calculation was performed, as recruitment was based on convenience sampling.

The survey was administered using Google Forms. The criterion of childbirth within the preceding 12 months was stated in the survey introduction; no separate screening questions or automated eligibility verification were implemented. The questionnaire did not use automated skip logic. All closed-ended analytical items were mandatory, while the final open-ended question was optional. Consequently, questionnaires could not be submitted with missing responses to the analytical items. No financial or non-financial incentives were offered. Detailed operational records concerning questionnaire views, starts, and potential duplicate submissions were not retained; therefore, completion rates and detailed participant-flow information could not be determined. Duplicate participation could not be entirely excluded.

All participants were informed about the purpose of the study, the voluntary nature of participation, anonymity, and the method of data processing before completing the questionnaire. Participants were required to indicate their electronic informed consent in Google Forms before proceeding to the questionnaire. No directly identifying information was collected.

### 2.3. Instrument

Data were collected with an investigator-developed questionnaire informed by a review of the literature on early-postpartum emotional experiences, breastfeeding difficulties, and social support [15,16,17,18,19,20,21,22,23,24,25,26,27,28,29]. The literature review informed three main thematic areas: early-postpartum emotional distress, including emotional symptoms occurring within the first 14 days postpartum [17,19,23,24,25]; breastfeeding-related difficulties and emotional burden [21,26,27]; and professional and social support, including support from midwives, community health nurses, and lactation consultants [15,16,28,29].

The questionnaire assessed sociodemographic and perinatal characteristics (age, parity, mode of birth, gestational age), infant feeding and breastfeeding difficulties (planning to breastfeed, presence of difficulties, type of difficulties), emotional burden related to breastfeeding difficulties, professional and emotional support, emotional symptoms during the first 14 days postpartum (tearfulness, mood changes, sadness, anxiety, insecurity in the maternal role), their perceived impact on daily functioning, and the perceived relationship between breastfeeding difficulties and emotional state. It was not a diagnostic instrument, and no clinical cut-off score was used.

Questionnaire development was additionally informed by interviews with 10 mothers, who discussed their experiences of early breastfeeding difficulties, the associated emotional burden, and the professional and personal support they had received or considered necessary. Their perspectives were used to refine the content and wording of the questionnaire items and to ensure that the questionnaire reflected issues relevant to mothers’ lived experiences.

The questionnaire was pilot tested in 223 participants for clarity and feasibility. Because no changes were made to items, response options, or scoring based on the pilot, these responses were included in the final analytic sample of 1378 participants. The pilot was not an independent validation sample.

The early-postpartum emotional symptom score consisted of five items asking how strongly, during the first 14 days after childbirth, mothers experienced frequent crying without a clear reason, mood changes, sadness or emptiness, anxiety or inner unrest, and insecurity about their ability to care for the infant. The five items were scored on a 5-point Likert scale from 1 (not at all) to 5 (very pronounced). The score was calculated as the mean of all items, with a range from 1 to 5, with higher values indicating more pronounced symptoms. All five items were needed to calculate the final score.

Breastfeeding difficulties were assessed using a yes/no item referring to the first 14 days postpartum, followed by a multiple-response item about difficulty types. Emotional burden attributed to breastfeeding difficulties was rated from 1 (not at all) to 5 (extremely). The H2 analysis was restricted to the 1093 women reporting breastfeeding difficulties, as the emotional-burden item specifically referred to those difficulties. We also examined the applicability of the breastfeeding-related emotional burden item among participants reporting exclusive formula feeding during the first seven days postpartum. Of the 85 participants in this group, 74 (87.1%) reported breastfeeding difficulties, whereas 11 (12.9%) did not. Therefore, exclusive formula feeding was not considered equivalent to the absence of breastfeeding difficulties. The H2 analysis included participants reporting breastfeeding difficulties irrespective of their feeding method during the first seven days postpartum.

Perceived emotional support in the early postpartum period was measured by a single item rated from 1 (not at all) to 5 (completely), not by a validated multidimensional scale. Because up to one year may have elapsed between childbirth and survey completion, each relevant question included a reminder asking participants to recall specifically their experiences during the first 14 days postpartum.

The complete questionnaire in Croatian, together with its English translation, is provided in Supplementary File S2.

### 2.4. Variables

The outcome was the early-postpartum emotional symptom score, calculated as the mean of five items (range 1–5).

The primary predictors were the presence of breastfeeding difficulties (0 = no, 1 = yes), emotional burden related to breastfeeding difficulties (1–5), and perceived emotional support (1–5). Based on the assessment of linearity, breastfeeding-related emotional burden was treated as a categorical variable (ratings 2–5 compared with rating 1) in the H2 and H3 regression models, whereas perceived emotional support was treated as a continuous predictor.

Covariates in adjusted models were maternal age, education, employment status, place of residence, parity, mode of birth, gestational age at birth, and rooming-in practice. Their selection reflects plausible maternal sociodemographic and perinatal associations with early postpartum experiences. Infant feeding method in the first seven days was excluded from the primary adjusted models because it may occur after breastfeeding difficulties and may therefore represent a downstream variable.

### 2.5. Statistical Analysis

Statistical analysis was performed using IBM SPSS Statistics, version 21 (IBM Corp., Armonk, NY, USA). Categorical variables are reported as frequencies and percentages; continuous or scale scores are reported as means and standard deviations or as medians and interquartile ranges. With multiple-choice questions, the use of participants as the denominator results in percentages of responses that may sum to more than 100%.

Internal consistency of the five-item score for emotional symptoms was estimated using Cronbach’s alpha, its 95% confidence interval, corrected item-total correlations, Cronbach’s alpha if item deleted, and McDonald’s omega total. Sampling adequacy and one-factor structure were explored using the Kaiser–Meyer–Olkin statistic, Bartlett’s test of sphericity, and one-factor principal axis factoring.

For the main comparisons, the Mann–Whitney U test evaluated differences in the emotional symptom score between participants with and without breastfeeding difficulties; the rank-biserial correlation and Hedges’ g were reported as effect sizes with 95% confidence intervals. Spearman’s rho was reported for associations of emotional burden and perceived emotional support with the symptom score.

For H1, unadjusted and covariate-adjusted ordinary least-squares regression models examined the association between breastfeeding difficulties (yes versus no) and the early-postpartum emotional symptom score (*N* = 1378). For H2, the adjusted analysis was restricted to women reporting breastfeeding difficulties (*n* = 1093) and included emotional burden as a categorical predictor (reference: rating 1), perceived emotional support as a numerical predictor, and covariates. For H3, a separate adjusted model was fitted in the full sample (*N* = 1378), including categorical emotional burden, numerical perceived emotional support, and the same covariates.

Adjusted models included maternal age, education, employment status, place of residence, parity, mode of birth, gestational age at birth, and rooming-in. Unstandardized regression coefficients (B), 95% confidence intervals, and model fit statistics were reported.

For H4, moderation was examined using a regression model including breastfeeding-difficulty status, perceived emotional support, and their interaction term. Unadjusted and covariate-adjusted models were fitted in the full sample (*N* = 1378). Conditional associations between breastfeeding difficulties and emotional symptom scores were estimated at low and high levels of perceived emotional support, defined as one standard deviation below and above the sample mean. A supplementary analysis examined the interaction using categorical rather than numerical emotional support.

Regression diagnostics included examination of residual distributions and plots, standardized residuals, variance inflation factors, tolerance, Cook’s distance, and Durbin–Watson statistics. The functional forms of emotional burden and perceived emotional support were assessed by comparing numerical and categorical specifications. Heteroskedasticity was assessed using auxiliary regressions of squared residuals, and additional analyses used sandwich-robust standard errors. Sensitivity analyses excluded potentially influential observations identified using Cook’s distance greater than 4/*n*, without excluding these observations from the primary analyses.

A further sensitivity analysis repeated the main tests after excluding participants who reported symptoms lasting longer than 14 days. All analyses used complete cases, with no missing data for variables included in the relevant analytic samples (*N* = 1378 for H1, H3, and H4; *n* = 1093 for H2). Two-sided *p*-values below 0.05 were considered statistically significant.

### 2.6. Ethical Considerations

The study was conducted in accordance with the principles of the Declaration of Helsinki. Participation was voluntary, and the questionnaire was completed anonymously. Participants were informed about the purpose of the study and the voluntary nature of participation before completing the questionnaire. No personally identifiable information was collected.

The study documentation was retrospectively reviewed by the Ethics Committee of the Faculty of Health Sciences, University of Split, after the completion of data collection. The Committee was informed that data collection had already been completed and, following its review, issued a decision confirming that the study had been conducted in accordance with applicable ethical principles (Class: 029-03/26-08/01; Reference No.: 2181-228-103/1-81) on 8 October 2026.

## 3. Results

### 3.1. Participant Characteristics

The final analytical sample included all 1378 participants. Participant characteristics are shown in Table 1.

The largest age group was 31–35 years (38.0%), followed by 26–30 years (29.6%). Most participants had completed or were attending graduate-level education (42.6%), were employed (54.8%), and lived in an urban area (69.3%). Slightly more than half of the participants were primiparous (53.3%). Most births occurred at term between 37 and 42 weeks of gestation (91.8%), and rooming-in was reported by 82.1% of participants.

### 3.2. Breastfeeding Intention, Feeding Practices, and Breastfeeding Difficulties

Most participants planned to breastfeed during pregnancy (91.0%). In the first seven days postpartum, 44.0% of infants were exclusively breastfed, 49.9% received breastfeeding with supplementation, and 6.2% were exclusively formula-fed. Overall, 93.8% of infants received breast milk either exclusively or in combination with supplementation during the first week. Breastfeeding difficulties during the first 14 days postpartum were reported by 79.3% of participants (Table 2).

In the total sample (*N* = 1378), the most frequently reported breastfeeding difficulties were sore or damaged nipples (46.7%), difficulties with infant latch (46.5%), pain during breastfeeding (36.3%), perceived insufficient milk supply (28.0%), and milk stasis or mastitis (14.2%) (Table 3). Multiple responses were permitted; therefore, percentages do not sum to 100%.

Emotional burden attributed to breastfeeding difficulties was rated as 5 (extreme) by 514 participants (37.3%), 4 by 290 (21.0%), 3 by 255 (18.5%), 2 by 126 (9.1%), and 1 (none) by 193 (14.0%). In the full sample, 58.3% selected ratings 4 or 5 on the breastfeeding-related emotional-burden item; because the item was completed by all participants, this full-sample distribution should be interpreted cautiously.

### 3.3. Professional and Emotional Support

The most frequently reported source of professional breastfeeding support was the community health nurse (50.5%), followed by midwives (15.9%) and lactation consultants (15.7%) (Table 4). However, 35.6% of participants reported no professional breastfeeding support. Only 30.8% of participants considered the breastfeeding support they received sufficient, while 39.9% considered it insufficient and 29.3% only partially sufficient.

A person to confide in after childbirth was consistently available to 954 participants (69.2%), occasionally available to 297 (21.6%), and unavailable to 127 (9.2%). The most frequently selected sources of emotional support were partners (82.7%), family members (52.1%), community health nurse (21.2%), and friends (15.5%).

**Table 4:** Professional and emotional support sources among participants during the early postpartum period (*N* = 1378; multiple responses permitted).

Support Variable	Category	*n*	%
Professional breastfeeding support	Community health nurse	696	50.5
	No support	491	35.6
	Midwife	219	15.9
	Lactation consultant	217	15.7
Perceived sufficient breastfeeding support	Yes	424	30.8
	Partially	404	29.3
	No	550	39.9
Emotional support	Partner	1139	82.7
	Family members	718	52.1
	Friends	213	15.5
	Community health nurse	292	21.2
	Midwife	36	2.6
	Other health professionals	36	2.6
	Nobody	81	5.9

Values are presented as *n* (%), with percentages calculated using the total sample (*N* = 1378). Multiple responses were permitted for professional and emotional support sources; therefore, percentages within these groups do not sum to 100%. Ten participants selected both “no professional support” and at least one professional support source; these responses were retained as originally recorded in the descriptive analysis. The perceived sufficiency of breastfeeding support was assessed using a single-response question.

### 3.4. Early-Postpartum Emotional Symptoms

The five-item early-postpartum emotional symptom score had a mean of 3.16 (SD = 1.10; possible range 1–5). At the item level, mean scores ranged from 2.98 for insecurity about the ability to care for the infant to 3.61 for mood changes (Table 5). Internal consistency was good (Cronbach’s alpha = 0.847, 95% CI 0.834–0.860; McDonald’s omega total = 0.853). Corrected item-total correlations ranged from 0.548 to 0.757. The KMO value was 0.816 and Bartlett’s test was significant, χ^2^ (10) = 3008.41, *p* < 0.001. A one-factor principal axis model produced loadings from 0.594 to 0.855 (Table 5). The adequacy of the data for factor analysis was supported by the KMO statistic and Bartlett’s test, while all five items showed positive and substantial loadings on the extracted factor. These findings provide preliminary support for an approximately unidimensional score in this sample, although they do not establish full construct validity or demonstrate that a one-factor solution is superior to alternative factor structures.

Regarding retrospectively reported symptom duration, 659 participants (47.8%) reported symptoms lasting longer than 14 days, 332 (24.1%) reported symptoms lasting 8–14 days, 150 (10.9%) reported symptoms lasting 3–7 days, 108 (7.8%) reported symptoms lasting less than 3 days, and 129 (9.4%) reported none of the listed feelings. Because symptom duration longer than 14 days extends beyond the conventional time frame of transient maternity blues, the primary outcome is described as an early-postpartum emotional symptom score rather than diagnosed baby blues. A sensitivity analysis excluding the 659 participants who reported symptoms lasting longer than 14 days was therefore performed.

The descriptive distributions of symptom-related impact on daily functioning and symptom duration are presented in Table 2. Sociodemographic and perinatal variables were treated as covariates in the primary adjusted models rather than examined as a series of separate unplanned subgroup comparisons.

### 3.5. Hypothesis Testing

Participants who reported breastfeeding difficulties had higher early-postpartum emotional symptom scores than those without breastfeeding difficulties (M = 3.28, SD = 1.07, median = 3.40, IQR = 2.40–4.00 vs. M = 2.72, SD = 1.10, median = 2.80, IQR = 1.80–3.40). The Mann–Whitney U test showed a statistically significant difference between the groups (U = 110,871, *p* < 0.001), with a rank-biserial correlation of 0.288 (95% CI 0.221–0.355) and Hedges’ g of 0.519 (95% CI 0.387–0.651). In a multivariable linear regression model with the early-postpartum emotional symptom score as the dependent variable and breastfeeding difficulties as the primary predictor, after adjustment for maternal age, education, employment status, place of residence, parity, mode of birth, gestational age at birth, and rooming-in, breastfeeding difficulties remained associated with a higher emotional symptom score (B = 0.490, 95% CI 0.350–0.631, *p* < 0.001; *N* = 1378; R^2^ = 0.079, adjusted R^2^ = 0.064). Therefore, H1 was supported.

Among participants reporting breastfeeding difficulties (*n* = 1093), greater emotional burden was positively associated with the early-postpartum emotional symptom score (Spearman’s ρ = 0.347, *p* < 0.001). In a multivariable linear regression model with the early-postpartum emotional symptom score as the dependent variable and breastfeeding-related emotional burden and perceived emotional support as the primary predictors, adjustment was made for maternal age, education, employment status, place of residence, parity, mode of birth, gestational age at birth, and rooming-in. Because the association between emotional burden and symptom scores deviated significantly from linearity (F(3, 1066) = 6.741, *p* < 0.001), emotional burden was modelled categorically, with rating 1 as the reference category. The highest burden rating (5) was associated with a higher emotional symptom score compared with rating 1 (B = 0.721, 95% CI 0.415–1.028, *p* < 0.001), whereas ratings 2–4 did not differ significantly from the reference category. The adjusted model explained 23.1% of the variance in emotional symptom scores (R^2^ = 0.231, adjusted R^2^ = 0.212). These findings provided support for H2, although the association was not linear across burden ratings.

Greater perceived emotional support was negatively associated with the early-postpartum emotional symptom score (Spearman’s ρ = −0.262, *p* < 0.001). In a separate multivariable linear regression model including the full sample and the predictors and covariates described above, each one-point increase in perceived emotional support was associated with a 0.241-point lower emotional symptom score (B = −0.241, 95% CI −0.287 to −0.195, *p* < 0.001; *N* = 1378; R^2^ = 0.224, adjusted R^2^ = 0.209). Therefore, H3 was supported.

To test H4, a moderation analysis was performed using a multivariable linear regression model with the early-postpartum emotional symptom score as the dependent variable and breastfeeding-difficulty status, perceived emotional support, and their interaction term as predictors. The interaction between breastfeeding difficulties and perceived emotional support was not statistically significant in the unadjusted model (B = −0.050, 95% CI −0.168 to 0.068, *p* = 0.403). It also remained non-significant in the model adjusted for maternal age, education, employment status, place of residence, parity, mode of birth, gestational age at birth, and rooming-in (B = −0.052, 95% CI −0.169 to 0.065, *p* = 0.381; *N* = 1378; R^2^ = 0.146, adjusted R^2^ = 0.131). The estimated difference in emotional symptom scores associated with breastfeeding difficulties was 0.526 points at low perceived support (95% CI 0.330–0.722) and 0.407 points at high perceived support (95% CI 0.222–0.592), defined as one standard deviation below and above the mean support score, respectively. However, because the interaction term was not statistically significant, these conditional estimates should not be interpreted as evidence of moderation. Therefore, H4 was not supported. Although the interaction was not statistically significant, its 95% confidence interval (−0.169 to 0.065) does not exclude potentially meaningful moderation effects; therefore, the result should not be interpreted as evidence of their absence.

Regression diagnostics were examined for all four adjusted models. Durbin–Watson statistics were 1.966, 1.989, 1.918, and 1.940 for H1–H4, respectively. Residual plots suggested no major departures from symmetry. Only one observation in the H2 model had a standardized residual exceeding an absolute value of 3, whereas no such observations were identified in H1, H3, or H4. The maximum Cook’s distance was 0.117 for H1 and 0.089 for H4 and remained below 1 in all four models. Multicollinearity diagnostics were generally acceptable across the regression models, although higher variance inflation factors were observed for some categorical indicators of emotional burden in H2 (maximum VIF = 7.320), likely reflecting the use of multiple indicator variables with a relatively small reference category. Assessment of linearity supported retaining perceived emotional support as a linear predictor, whereas emotional burden was modeled categorically because of evidence of nonlinearity. Auxiliary tests indicated heteroskedasticity in H1 (*p* = 0.042) and H4 (*p* = 0.002), but not in H2 (*p* = 0.466) or H3 (*p* = 0.300). Additional analyses using sandwich-robust standard errors and sensitivity analyses excluding potentially influential observations did not change the principal findings.

Complete regression coefficients for the adjusted and unadjusted models are provided in Supplementary File S1 (Tables S1–S5). A summary of the primary inferential analyses for hypotheses H1–H4 is presented in Table 6.

A sensitivity analysis excluding participants who reported symptoms lasting longer than 14 days (*n* = 719) yielded findings consistent with the primary analyses. Breastfeeding-difficulty groups remained significantly different (Mann–Whitney U = 39,847.5, *p* < 0.001), and breastfeeding difficulties remained positively associated with the emotional symptom score after adjustment (B = 0.342, 95% CI 0.169 to 0.516, *p* < 0.001). Among participants reporting breastfeeding difficulties (*n* = 524), emotional burden remained positively correlated with the symptom score (Spearman’s ρ = 0.328, *p* < 0.001), with a significant adjusted association for the highest burden rating compared with the lowest (B = 0.914, 95% CI 0.493 to 1.336, *p* < 0.001). Perceived emotional support remained negatively associated with the symptom score (Spearman’s ρ = −0.208, *p* < 0.001; adjusted B = −0.195, 95% CI −0.266 to −0.124, *p* < 0.001). The adjusted breastfeeding-difficulty × perceived-emotional-support interaction remained non-significant (B = −0.097, 95% CI −0.250 to 0.055, *p* = 0.211; R^2^ = 0.126).

## 4. Discussion

### 4.1. Study Results

This study examined the associations of breastfeeding difficulties, breastfeeding-related emotional burden, and perceived emotional support with retrospectively reported early-postpartum emotional symptoms. Breastfeeding difficulties and greater emotional burden were associated with higher symptom scores, whereas greater perceived emotional support was associated with lower symptom scores. These associations remained after adjustment for maternal and perinatal covariates. In contrast, the breastfeeding-difficulty × perceived-emotional-support interaction was not statistically significant, and the data therefore did not support H4.

Given the cross-sectional, retrospective design and the use of an investigator-developed non-diagnostic symptom score, the findings should be interpreted as associations rather than as evidence of causal effects or diagnosed maternity blues.

In this convenience sample, 79.3% of respondents reported breastfeeding difficulties during the first 14 days postpartum, most commonly sore or damaged nipples, difficulty with infant latch, and pain during breastfeeding. These findings are consistent with the clinical literature describing nipple trauma, engorgement, pain, milk-supply concerns, and latch problems as common challenges during early lactation [8,9]. Review evidence also indicates that difficult or distressing breastfeeding experiences can carry a substantial emotional burden for mothers [30]. Because recruitment was online and self-selected, however, the 79.3% figure should be interpreted as a characteristic of this sample rather than as a population prevalence estimate.

Participants reporting breastfeeding difficulties had higher early-postpartum emotional symptom scores than participants without such difficulties, and the association remained after covariate adjustment. The magnitude of the between-group difference was also supported by the reported rank-biserial correlation and Hedges’ g. Although the outcome in the present study is not equivalent to postpartum depression, the direction of the association is consistent with studies linking breastfeeding difficulties or unsatisfactory breastfeeding experiences with poorer postpartum emotional outcomes [21,26,27,30]. Chaput et al., for example, prospectively examined breastfeeding difficulties and support in relation to postpartum depressive symptoms [26]. The present findings are consistent with and complement the literature by showing a similar association with a broader, non-diagnostic early-postpartum emotional symptom score, while not implying that breastfeeding difficulties cause those symptoms.

Among women reporting breastfeeding difficulties, greater emotional burden was positively associated with early-postpartum emotional symptom scores, although the adjusted association was not adequately represented by a single linear slope. In the adjusted analysis, the highest burden rating (5 compared with 1) was associated with higher symptom scores, whereas comparisons for ratings 2–4 were not statistically significant. This finding highlights the importance of the subjective emotional experience of breastfeeding difficulties, which may not be fully captured by the mere presence or absence of a specific feeding difficulty. Pain, perceived failure to meet feeding expectations, uncertainty about milk supply, and distress related to the milk-ejection or feeding experience can influence how breastfeeding challenges are experienced emotionally [30]. The present findings therefore suggest that both the presence of breastfeeding difficulties and the perceived emotional burden associated with these experiences are relevant dimensions of early postpartum breastfeeding experiences. Given the cross-sectional and retrospective study design, the temporal direction of these associations cannot be established.

Greater perceived emotional support was associated with lower early-postpartum emotional symptom scores in both the bivariate and adjusted analyses. This is consistent with a broader body of the postpartum literature in which social support is associated with more favourable maternal psychosocial outcomes [16]. Evidence from breastfeeding research also suggests that the role of support depends on both the type of difficulty and the form of support provided; Page et al. reported context-dependent buffering of breastfeeding problems in relation to breastfeeding cessation [31]. Studies focused on postpartum mental health have similarly reported associations between greater support and lower postpartum depressive or anxiety symptoms [32,33,34], while research on infant-feeding experiences has distinguished the contributions of practical and emotional support [35]. These studies support the relevance of perceived support, but their outcomes and measurement approaches differ from the early-postpartum emotional symptom score used here.

Importantly, the present moderation analysis did not show that perceived emotional support significantly altered the association between breastfeeding difficulties and the emotional symptom score. The adjusted breastfeeding-difficulty × perceived-emotional-support interaction was not statistically significant, and H4 was therefore not supported. This distinction is important because a main association between greater support and lower symptom scores does not, by itself, demonstrate statistical moderation or a buffering effect. The findings of Page et al. [31] provide evidence of buffering in relation to breastfeeding continuation, but that outcome is different from maternal emotional symptoms; the current data do not justify extending that buffering interpretation to the present outcome.

The duration findings further reinforce the need for cautious interpretation of the outcome construct. Almost half of respondents reported that the listed emotional symptoms lasted longer than 14 days, which extends beyond the conventional time frame generally used to describe transient maternity blues [17,19,23]. For this reason, the study outcome is appropriately described as an early-postpartum emotional symptom score rather than as a diagnostic measure of maternity blues. The sensitivity analysis excluding participants who reported symptoms lasting longer than 14 days produced the same overall pattern: breastfeeding difficulties remained associated with higher scores, emotional burden remained positively associated with the score, perceived emotional support remained negatively associated with the score, and the interaction remained non-significant. These findings provide some support for the robustness of the main pattern of associations, while not resolving the limitations inherent in retrospective symptom recall.

Comparison with the wider literature should also take account of differences in outcome definition. Much of the existing evidence on breastfeeding and maternal mental health concerns postpartum depression or anxiety rather than transient early-postpartum emotional symptoms [21,22,26,27,32,33,34]. These clinical or screening-based constructs overlap with some of the symptoms included in the present five-item score, but they are not interchangeable with it. The current findings should therefore be viewed as complementary to, rather than direct estimates of, the associations reported in studies using validated postpartum depression or anxiety instruments.

Taken together, the results suggest that breastfeeding difficulties, the emotional burden attached to those difficulties, and perceived emotional support represent related but distinct dimensions of early postpartum experience. Breastfeeding difficulties and emotional burden were associated with higher emotional symptom scores, whereas perceived emotional support was associated with lower scores. The lack of a significant interaction indicates that support should not be described as statistically buffering the association between breastfeeding difficulties and symptoms in this dataset. Prospective studies conducted close to childbirth, using validated measures and repeated assessments, are needed to clarify temporality and to determine whether specific forms of breastfeeding or emotional support modify subsequent maternal emotional outcomes.

### 4.2. Implications for Practice

The findings suggest a need for a clinically integrated approach involving breastfeeding difficulty and maternal emotional wellbeing. Women who report early breastfeeding difficulties or significant emotional burden may need increased attention to their emotional wellbeing, and women who report low levels of perceived support could benefit from a discussion of potential sources of support. Community health nurses, midwives, lactation consultants, and other postpartum-care professionals are well positioned to recognize concurrent challenges with breastfeeding and emotional wellbeing and to facilitate appropriate support. This interpretation is consistent with broader recommendations for supportive and woman-centred maternity care [28,29]. However, because the present study did not evaluate an intervention or a diagnostic screening strategy, these implications should be regarded as considerations for supportive care rather than as evidence that any specific intervention would improve maternal mental health outcomes.

### 4.3. Strengths and Limitations

The strengths of this study include the relatively large sample, the simultaneous examination of breastfeeding difficulties, breastfeeding-related emotional burden, and perceived emotional support, and the assessment of the emotional symptom score’s internal consistency and approximate unidimensionality. Additional strengths include the use of covariate-adjusted regression analyses, effect-size estimates, regression diagnostics, and sensitivity analysis. The detailed reporting of the questionnaire and statistical analyses in the supplementary materials further supports the transparency and reproducibility of the study.

Several limitations should be acknowledged. First, the cross-sectional design prevents causal conclusions. Reverse causation cannot be excluded, as maternal emotional symptoms may have influenced the perception and retrospective reporting of breastfeeding difficulties and their associated emotional burden.

Second, women were eligible up to one year after childbirth but retrospectively reported experiences from the first 7–14 days postpartum. Recall bias and retrospective reinterpretation may therefore be important concerns, particularly for transient emotional experiences, while mood-congruent recall may have further influenced participants’ responses. Furthermore, the exact interval between childbirth and survey completion was not collected, precluding an assessment of its potential influence on reporting accuracy. Consequently, the findings should be interpreted as associations involving retrospectively reported early-postpartum emotional symptoms rather than contemporaneously assessed emotional states. Although the duration-restricted sensitivity analysis yielded similar findings, it does not eliminate recall bias, as all participants reported their experiences retrospectively.

Third, recruitment relied on a convenience online sample distributed mainly through social-media groups and private messaging channels. The sampling frame and response denominator were unknown, and self-selection, digital-access and coverage bias may have occurred. Women with particularly salient breastfeeding or emotional experiences may have been more motivated to participate, and the sample should not be considered representative of postpartum women in Croatia.

Fourth, the five-item early-postpartum emotional symptom score was investigator-developed and non-diagnostic. Although internal consistency and approximate one-dimensionality were supported in this sample, these findings do not establish full construct or diagnostic validity. Nearly half of respondents reported symptoms lasting longer than 14 days, further indicating that the measured construct may extend beyond transient maternity blues. Furthermore, breastfeeding-related emotional burden and the emotional symptom score were assessed using the same questionnaire and may capture partly overlapping subjective emotional experiences. Common-method bias and content overlap may therefore have contributed to the observed associations.

Fifth, perceived emotional support was assessed using a single item rather than a validated multidimensional social-support instrument, limiting measurement precision and preventing assessment of its internal reliability.

Finally, although the regression models adjusted for several maternal and perinatal covariates, residual confounding remains possible because potentially relevant factors such as prior mental-health history, pregnancy or birth complications, pain, sleep, socioeconomic circumstances, and postpartum time at survey were not fully captured or adjusted for. Future prospective studies should assess women close to childbirth, use validated measures of postpartum emotional symptoms and social support, and evaluate temporal changes and potential mechanisms longitudinally.

## 5. Conclusions

In this convenience sample, breastfeeding difficulties during the first 14 days postpartum and greater breastfeeding-related emotional burden were associated with higher retrospectively reported early-postpartum emotional symptom scores, whereas greater perceived emotional support was associated with lower scores. These associations remained statistically significant after adjustment for covariates. However, the interaction between breastfeeding difficulties and perceived emotional support was not statistically significant. Therefore, no statistical evidence was found that perceived emotional support moderated the association between breastfeeding difficulties and emotional symptoms in this sample.

The findings suggest that breastfeeding experiences, breastfeeding-related emotional burden, and perceived emotional support should be considered together when assessing postpartum wellbeing. Given the retrospective, cross-sectional design, convenience sampling, and reliance on a non-diagnostic symptom score and a single-item measure of perceived emotional support, the findings should not be interpreted as causal or generalized to estimate the prevalence of maternity blues among postpartum women in Croatia. Prospective longitudinal studies using validated instruments are needed to clarify temporal relationships and determine whether integrated breastfeeding and emotional-support interventions improve maternal outcomes.

## Figures and Tables

**Table 1:** Sociodemographic and perinatal characteristics of participants (*N* = 1378).

Characteristic	Category	*n* (%)
Age (years)	≤20	4 (0.3)
	21–25	96 (7.0)
	26–30	408 (29.6)
	31–35	523 (38.0)
	36–40	272 (19.7)
	41–45	72 (5.2)
	>45	3 (0.2)
Education	Primary school	5 (0.4)
	Secondary school	392 (28.4)
	College/undergraduate study	283 (20.5)
	Graduate study	587 (42.6)
	Postgraduate study	111 (8.1)
Employment status	Employed	755 (54.8)
	Unemployed	97 (7.0)
	Student	8 (0.6)
	Maternity/parental leave	518 (37.6)
Place of residence	Urban	955 (69.3)
	Suburban	250 (18.1)
	Rural	173 (12.6)
Parity	First birth	735 (53.3)
	Previous birth(s)	643 (46.7)
Mode of birth	Vaginal birth	669 (48.5)
	Vaginal birth with episiotomy	315 (22.9)
	Caesarean section	394 (28.6)
Gestational age at birth	<37 weeks	77 (5.6)
	37–42 weeks	1265 (91.8)
	>42 weeks	36 (2.6)
Rooming-in	Yes	1131 (82.1)
	No	247 (17.9)

Values are presented as *n* (%), with percentages calculated using the total sample (*N* = 1378). No missing values were recorded for the variables presented. Education and mode-of-birth categories correspond to the original questionnaire response options. Mode-of-birth categories were recorded as mutually exclusive responses, with vaginal birth with episiotomy included as a separate response category.

**Table 2:** Breastfeeding, support, and symptom-related characteristics (*N* = 1378).

Variable	Category	*n* (%)
Planned breastfeeding	Yes	1254 (91.0)
	No	48 (3.5)
	Had not considered it	76 (5.5)
Feeding in first 7 days	Exclusive breastfeeding	606 (44.0)
	Breastfeeding with supplementation	687 (49.9)
	Exclusive formula feeding	85 (6.2)
Breastfeeding difficulties in first 14 days	Yes	1093 (79.3)
Impact of feelings on daily functioning	No listed feelings	43 (3.1)
	None	130 (9.4)
	Mild	373 (27.1)
	Moderate	495 (35.9)
	Substantial	337 (24.5)
Reported symptom duration	No listed feelings	129 (9.4)
	<3 days	108 (7.8)
	3–7 days	150 (10.9)
	8–14 days	332 (24.1)
	>14 days	659 (47.8)

Values are presented as *n* (%), with percentages calculated using the total sample (*N* = 1378). No missing values were recorded for the variables presented. “No listed feelings” represents a separate response option for the questions on daily functioning and symptom duration. Percentages may not sum to 100% due to rounding.

**Table 3:** The five most frequently reported breastfeeding difficulties during the first 14 days postpartum in the total sample (*N* = 1378).

Breastfeeding Difficulty	*n*	%
Sore or damaged nipples	643	46.7
Difficulties with infant latch	641	46.5
Pain during breastfeeding	500	36.3
Perceived insufficient milk supply	386	28.0
Milk stasis or mastitis	195	14.2

Multiple responses were permitted. Percentages were calculated using the total sample (*N* = 1378) and reflect the individual breastfeeding difficulties selected by participants. Percentages therefore do not sum to 100%.

**Table 5:** Item-level descriptive statistics, reliability, and factor analysis results for the five-item early-postpartum emotional symptom score.

Item	Mean	SD	Corrected Item-Total Correlation	One-Factor PAF Loading	Cronbach’s Alpha If Item Deleted
Frequent crying without a clear reason	3.10	1.419	0.621	0.684	0.826
Mood changes	3.61	1.176	0.644	0.705	0.822
Sadness or emptiness	3.01	1.465	0.757	0.855	0.787
Anxiety or inner unrest	3.10	1.426	0.730	0.809	0.796
Insecurity about ability to care for infant	2.98	1.468	0.548	0.594	0.846

*N* = 1378 for all items; no missing values. All items were scored from 1 (not at all) to 5 (very pronounced). SD = standard deviation; PAF = principal axis factoring. Overall Cronbach’s alpha = 0.847 (95% CI 0.834–0.860); McDonald’s omega total = 0.853; KMO = 0.816; Bartlett’s χ^2^ (10) = 3008.41, *p* < 0.001.

**Table 6:** Primary inferential analyses of the early-postpartum emotional symptom score.

	Analysis/Predictor	Estimate	95% CI	*p*-Value	Conclusion
H1	Breastfeeding difficulties, rank-biserial *r*	0.288	0.221 to 0.355	<0.001	Supported
	Breastfeeding difficulties, adjusted B	0.490	0.350 to 0.631	<0.001	
H2	Emotional burden, Spearman’s ρ (restricted sample)	0.347	0.291 to 0.401	<0.001	Supported
	Emotional burden, rating 5 vs. 1, adjusted B (restricted sample)	0.721	0.415 to 1.028	<0.001	
H3	Perceived emotional support, Spearman’s ρ	−0.262	−0.311 to −0.212	<0.001	Supported
	Perceived emotional support, adjusted B	−0.241	−0.287 to −0.195	<0.001	
H4	Breastfeeding difficulties × perceived emotional support, adjusted B	−0.052	−0.169 to 0.065	0.381	Not supported

B = unstandardized regression coefficient; CI = confidence interval; ρ = Spearman’s rank correlation coefficient. Adjusted models included maternal age, education, employment status, place of residence, parity, mode of birth, gestational age, and rooming-in. H2 analyses were restricted to participants reporting breastfeeding difficulties (*n* = 1093); H1, H3, and H4 analyses included the full sample (*N* = 1378). Emotional burden was modeled categorically in H2 and H3, with rating 1 as the reference category. The H2 adjusted coefficient shown represents rating 5 versus rating 1. Complete regression results, including coefficients for all predictors and covariates, are provided in the supplementary regression tables (Supplementary Tables S1–S5).

## Data Availability

The data presented in this study are available from the corresponding author upon reasonable request. The data are not publicly available due to privacy and ethical restrictions related to the protection of participants’ confidentiality.

## Supplementary Materials

The following supporting information can be downloaded at https://www.mdpi.com/article/10.3390/healthcare14213680/s1: Supplementary File S1: Supplementary Regression Results (Tables S1–S5); Supplementary File S2: Questionnaire—Original Croatian Version and English Translation.
