# Supplementary material for: Association of Breastfeeding Difficulties and Perceived Emotional Support with Retrospectively Reported Early-Postpartum Emotional Symptoms: A Cross-Sectional Study in Croatia
Source: Healthcare (Basel). 2026 Oct 30;14(21):3680. doi: 10.3390/healthcare14213680 (PMC13763558; doi:10.3390/healthcare14213680)
Supplement: Supplementary file 1 [file healthcare-14-03680-s001.zip › healthcare-4472501-supplementary.pdf]

healthcare

MDPI

Supplementary File S1

Supplementary regression results —adjusted and unadjusted models

Outcome: five-item early postpartum emotional symptom score (1–5).

Table S1. Multivariable linear regression analysis of the association between breastfeeding difficulties and early postpartum emotional symptom scores (H1).

Predictor / category comparison	B	95% CI	p-value
Breastfeeding difficulties: yes (ref: no)	0.490	0.350 to 0.631	<0.001
Maternal age (ref: 31–35)			
≤20	−0.442	-1.503 to 0.619	0.414
21–25	0.080	−0.170 to 0.330	0.531
26–30	0.026	−0.117 to 0.169	0.723
36–40	0.044	−0.114 to 0.202	0.589
41–45	0.029	−0.235 to 0.293	0.828
>45	−0.407	-1.627 to 0.813	0.513
Education (ref: graduate)			
primary	0.431	−0.512 to 1.375	0.370
secondary	0.095	−0.050 to 0.241	0.199
college/undergraduate	0.079	−0.076 to 0.235	0.319
postgraduate	−0.143	−0.362 to 0.077	0.202
Employment (ref: employed)			
unemployed	−0.249	−0.480 to −0.018	0.034
student	0.721	−0.042 to 1.484	0.064
maternity leave	−0.003	−0.127 to 0.120	0.957
Place of residence (ref: urban)			
rural	−0.066	−0.242 to 0.110	0.463
suburban	−0.055	−0.207 to 0.098	0.481
Parity (ref: previous birth(s))			
first birth	0.201	0.077 to 0.324	0.001
Mode of birth (ref: vaginal)			
vaginal birth with episiotomy	0.226	0.080 to 0.373	0.002
caesarean	0.185	0.044 to 0.326	0.010
Gestational age (ref: 37–42 weeks)			
<37	0.052	−0.201 to 0.306	0.686
>42	0.268	−0.087 to 0.623	0.139
Rooming-in (ref: no)			
yes	−0.140	−0.300 to 0.019	0.084

Note: B = unstandardized regression coefficient; CI = confidence interval; ref = reference category. N = 1,378. The model was adjusted for maternal age, education, employment status, place of residence, parity, mode of birth, gestational age at birth, and rooming-in. $R^2 = 0.079$; adjusted $R^2 = 0.064$; $F(22, 1355) = 5.301$, $p < 0.001$.

 https://doi.org/10.3390/healthcare14213680

Table S2. Multivariable linear regression analysis of the association between breastfeeding-related emotional burden and early postpartum emotional symptom scores (H2).

Predictor / category comparison	B	95% CI	p-value
Emotional burden (ref: rating 1)			
rating 2	−0.315	−0.669 to 0.038	0.081
rating 3	−0.025	−0.345 to 0.296	0.878
rating 4	0.262	−0.053 to 0.576	0.103
rating 5	0.721	0.415 to 1.028	<0.001
Perceived emotional support (per 1-point increase)	−0.244	−0.295 to −0.193	<0.001
Maternal age (ref: 31–35)			
≤20	−0.130	−1.231 to 0.971	0.817
21–25	0.086	−0.171 to 0.344	0.512
26–30	0.071	−0.071 to 0.214	0.326
36–40	0.013	−0.148 to 0.173	0.875
41–45	−0.019	−0.284 to 0.245	0.885
>45	−0.415	−1.514 to 0.684	0.458
Education (ref: graduate)			
primary	0.446	−0.501 to 1.394	0.356
secondary	0.103	−0.042 to 0.248	0.164
college/undergraduate	0.013	−0.143 to 0.170	0.867
postgraduate	−0.170	−0.396 to 0.056	0.140
Employment (ref: employed)			
unemployed	−0.306	−0.554 to −0.059	0.015
student	0.770	0.080 to 1.460	0.029
maternity leave	−0.091	−0.215 to 0.034	0.152
Place of residence (ref: urban)			
rural	−0.136	−0.314 to 0.043	0.136
suburban	−0.029	−0.184 to 0.126	0.715
Parity (ref: previous birth(s))			
first birth	0.212	0.089 to 0.335	<0.001
Mode of birth (ref: vaginal)			
vaginal birth with episiotomy	0.148	0.002 to 0.294	0.047
caesarean	0.164	0.022 to 0.306	0.024
Gestational age (ref: 37–42 weeks)			
<37	−0.030	−0.282 to 0.223	0.816
>42	0.129	−0.239 to 0.497	0.493
Rooming-in (ref: no)			
yes	−0.085	−0.241 to 0.071	0.283

Note: B = unstandardized regression coefficient; CI = confidence interval; ref = reference category. n = 1,093 women reporting breastfeeding difficulties. $R^2 = 0.231$; adjusted $R^2 = 0.212$; $F(26, 1066) = 12.288$, $p < 0.001$.

https://doi.org/10.3390/healthcare14213680

Table S3. Multivariable linear regression analysis of the association between perceived emotional support and early postpartum emotional symptom scores (H3).

Predictor / category comparison	B	95% CI	p-value
Emotional burden (ref: rating 1)			
rating 2	−0.084	−0.305 to 0.137	0.457
rating 3	0.170	−0.014 to 0.354	0.070
rating 4	0.411	0.232 to 0.591	<0.001
rating 5	0.870	0.704 to 1.035	<0.001
Perceived emotional support (per 1-point increase)	−0.241	−0.287 to −0.195	<0.001
Maternal age (ref: 31–35)			
≤20	−0.176	−1.153 to 0.801	0.724
21–25	0.197	−0.033 to 0.428	0.094
26–30	0.088	−0.044 to 0.220	0.190
36–40	0.037	−0.108 to 0.183	0.616
41–45	−0.015	−0.258 to 0.229	0.905
>45	−0.340	−1.463 to 0.782	0.552
Education (ref: graduate)			
primary	0.467	−0.402 to 1.337	0.292
secondary	0.070	−0.064 to 0.204	0.305
college/undergraduate	0.019	−0.124 to 0.162	0.795
postgraduate	−0.137	−0.339 to 0.065	0.185
Employment (ref: employed)			
unemployed	−0.314	−0.526 to −0.102	0.004
student	0.735	0.032 to 1.438	0.040
maternity leave	−0.061	−0.175 to 0.053	0.293
Place of residence (ref: urban)			
rural	−0.012	−0.174 to 0.150	0.887
suburban	−0.034	−0.174 to 0.107	0.637
Parity (ref: previous birth(s))			
first birth	0.166	0.052 to 0.280	0.004
Mode of birth (ref: vaginal)			
vaginal birth with episiotomy	0.178	0.043 to 0.313	0.010
caesarean	0.232	0.102 to 0.362	<0.001
Gestational age (ref: 37–42 weeks)			
<37	−0.020	−0.253 to 0.214	0.869
>42	0.155	−0.172 to 0.482	0.352
Rooming-in (ref: no)			
yes	−0.061	−0.208 to 0.086	0.413

Note: B = unstandardized regression coefficient; CI = confidence interval; ref = reference category. N = 1,378. $R^2 = 0.224$; adjusted $R^2 = 0.209$; $F(26, 1351) = 14.971$, $p < 0.001$.

Table S4. Multivariable linear regression analysis of the breastfeeding difficulties × perceived emotional support interaction and early postpartum emotional symptom scores (H4).

Predictor / category comparison	B	95% CI	p-value
Breastfeeding difficulties (ref: no)			
yes	0.656	0.203 to 1.109	0.005
Perceived emotional support (per 1 point)	−0.211	−0.315 to −0.107	<0.001
Breastfeeding difficulties × perceived emotional support	−0.052	−0.169 to 0.065	0.381
Maternal age (ref: 31–35)			
≤20	−0.453	−1.477 to 0.570	0.385
21–25	0.101	−0.140 to 0.342	0.410
26–30	0.044	−0.094 to 0.183	0.528
36–40	0.033	−0.119 to 0.185	0.672
41–45	0.005	−0.249 to 0.260	0.966
>45	−0.487	−1.663 to 0.688	0.416
Education (ref: graduate)			
primary	0.363	−0.546 to 1.273	0.434
secondary	0.047	−0.093 to 0.188	0.508
college/undergraduate	0.052	−0.098 to 0.202	0.500
postgraduate	−0.085	−0.297 to 0.127	0.434
Employment (ref: employed)			
unemployed	−0.293	−0.515 to −0.070	0.010
student	0.825	0.090 to 1.561	0.028
maternity leave	0.002	−0.117 to 0.121	0.970
Place of residence (ref: urban)			
rural	−0.023	−0.193 to 0.146	0.786
suburban	−0.050	−0.197 to 0.097	0.502
Parity (ref: previous birth(s))			
first birth	0.218	0.099 to 0.337	<0.001
Mode of birth (ref: vaginal)			
vaginal birth with episiotomy	0.209	0.068 to 0.351	0.004
caesarean	0.213	0.077 to 0.349	0.002
Gestational age (ref: 37–42 weeks)			
<37	−0.004	−0.249 to 0.240	0.972
>42	0.236	−0.107 to 0.579	0.177
Rooming-in (ref: no)			
yes	−0.114	−0.267 to 0.040	0.147

Note**:** B = unstandardized regression coefficient; CI = confidence interval; ref = reference category. N = 1,378. $R^2 = 0.146$; adjusted $R^2 = 0.131$; $F(24, 1353) = 9.665$, $p < 0.001$. The interaction term used uncentred breastfeeding-difficulty status and numerical perceived emotional support; the main effects are conditional at zero values of the interacting variable.

https://doi.org/10.3390/healthcare14213680

Table S5. Unadjusted linear regression analyses of the associations between breastfeeding difficulties, breastfeeding-related emotional burden, perceived emotional support, and early postpartum emotional symptom scores (H1–H4).

Predictor / category comparison	B	95% CI	p-value
**H1. Breastfeeding difficulties (N = 1,378)**			
Breastfeeding difficulties (ref: no)			
yes	0.558	0.418 to 0.699	<0.001
**H2. Breastfeeding-related emotional burden (n = 1,093)**			
Emotional burden (ref: rating 1)			
rating 2	−0.402	−0.770 to −0.034	0.032
rating 3	−0.043	−0.376 to 0.290	0.801
rating 4	0.273	−0.053 to 0.600	0.101
rating 5	0.732	0.416 to 1.048	<0.001
**H3. Emotional burden and perceived emotional support (N = 1,378)**			
Emotional burden (ref: rating 1)			
rating 2	−0.043	−0.266 to 0.180	0.705
rating 3	0.193	0.008 to 0.379	0.041
rating 4	0.462	0.282 to 0.643	<0.001
rating 5	0.928	0.764 to 1.093	<0.001
Perceived emotional support (per 1 point)	−0.237	−0.283 to −0.190	<0.001
**H4. Breastfeeding difficulties × perceived emotional support (N = 1,378)**			
Breastfeeding difficulties (ref: no)			
yes	0.719	0.264 to 1.173	0.002
Perceived emotional support (per 1 point)	−0.207	−0.312 to −0.103	<0.001
Interaction: difficulty × emotional support	−0.050	−0.168 to 0.068	0.403

Note: B = unstandardized regression coefficient; CI = confidence interval; ref = reference category. Unadjusted models included the predictors specified for each hypothesis without adjustment for maternal or perinatal covariates. Model fit statistics were as follows: H1 (N = 1,378), $R^2 = 0.042$, adjusted $R^2 = 0.042$, $F(1, 1376) = 60.892$, $p < 0.001$; H2 (n = 1,093), $R^2 = 0.128$, adjusted $R^2 = 0.125$, $F(4, 1088) = 40.062$, $p < 0.001$; H3 (N = 1,378), $R^2 = 0.191$, adjusted $R^2 = 0.188$, $F(5, 1372) = 64.751$, $p < 0.001$; H4 (N = 1,378), $R^2 = 0.108$, adjusted $R^2 = 0.106$, $F(3, 1374) = 55.528$, $p < 0.001$. Full covariate-adjusted regression results are presented in Tables S1–S4.

https://doi.org/10.3390/healthcare14213680

Supplementary File S2

Questionnaire: Original Croatian Version and English Translation

PART A | ORIGINAL CROATIAN QUESTIONNAIRE

ANKETA O DOJENJU I EMOCIONALNIM POTEŠKOĆAMA

Poštovana sudionice,

ova anketa dio je istraživanja o povezanosti poteškoća s dojenjem i emocionalnih poteškoća u ranom postpartalnom razdoblju u Republici Hrvatskoj.

U istraživanju mogu sudjelovati žene u dobi od 18 ili više godina koje su rodile unutar posljednjih 12 mjeseci. Sudjelovanje u istraživanju u potpunosti je dobrovoljno i anonimno, a prikupljeni podaci koristit će se isključivo u znanstvene svrhe. U upitniku nema točnih ni netočnih odgovora. Molimo Vas da se što iskrenije prisjetite razdoblja nakon poroda te svojih osjećaja, iskustava i potreba tijekom tog razdoblja.

Ispunjavanje ankete traje približno 7–10 minuta. Ako želite sudjelovati u ovom istraživanju, molimo Vas da označite elektronički pristanak za sudjelovanje. U bilo kojem trenutku možete odustati od istraživanja.

Zahvaljujemo Vam na vremenu, iskrenosti i sudjelovanju u ovom istraživanju.

I. OPĆI PODACI

Dob (u godinama): ___

Razina obrazovanja:

☐ Osnovna škola ☐ Srednja škola
☐ Viša škola / preddiplomski studij ☐ Diplomski studij
☐ Poslijediplomski studij

Status zaposlenja:

☐ Zaposlena ☐ Nezaposlena
☐ Studentica ☐ Rodiljni/roditeljski dopust

Mjesto stanovanja:

☐ Grad ☐ Prigradsko naselje ☐ Ruralno područje

II. PODACI O PORODU I NOVOROĐENČETU

Je li ovo Vaš prvi porod? ☐ Da ☐ Ne

Ako imate starije dijete jeste li ga dojili? ☐ Da ☐ Ne

Način poroda:

☐ Vaginalni porod ☐ Carski rez
☐ Vaginalni porod uz epiziotomiju

Tjedan trudnoće u kojem je porod dovršen: ___

Je li dijete nakon poroda bilo smješteno uz Vas (rooming-in)? ☐ Da ☐ Ne

III. DOJENJE

Jeste li planirali dojiti tijekom trudnoće? ☐ Da ☐ Ne ☐ Nisam razmišljala o tome

Način prehrane djeteta u prvih 7 dana nakon poroda:

☐ Isključivo dojenje ☐ Dojenje uz nadomjestak

☐ Isključivo adaptirano mlijeko

Jeste li imali poteškoće s dojenjem u prvih 14 dana nakon poroda?

Napomena: Pri odgovaranju na ovo pitanje molimo Vas da se prisjetite razdoblja nakon poroda te odgovorite na temelju svojih osjećaja, iskustava i potreba u tom razdoblju.

☐ Da ☐ Ne

Ako DA, označite koje (moguće više odgovora):

Napomena: Pri odgovaranju na ovo pitanje molimo Vas da se prisjetite razdoblja nakon poroda te odgovorite na temelju svojih osjećaja, iskustava i potreba u tom razdoblju.

☐ Bolne ili oštećene bradavice ☐ Poteškoće s prihvatom dojke

☐ Nedostatak mlijeka ☐ Zastoj mlijeka / mastitis

☐ Bol pri dojenju ☐ Drugo: ___________

Koliko su Vas poteškoće s dojenjem emocionalno opterećivale? (1 – nimalo, 5 – izuzetno)

Napomena: Pri odgovaranju na ovo pitanje molimo Vas da se prisjetite razdoblja nakon poroda te odgovorite na temelju svojih osjećaja, iskustava i potreba u tom razdoblju.

☐ 1 ☐ 2 ☐ 3 ☐ 4 ☐ 5

Jeste li imali stručnu podršku u dojenju?

Napomena: Pri odgovaranju na ovo pitanje molimo Vas da se prisjetite razdoblja nakon poroda te odgovorite na temelju svojih osjećaja, iskustava i potreba u tom razdoblju.

☐ Da, primalja ☐ Da, patronažna sestra

☐ Da, savjetnica za dojenje ☐ Ne

Smatrate li da ste dobili dovoljnu podršku za dojenje?

Napomena: Pri odgovaranju na ovo pitanje molimo Vas da se prisjetite razdoblja nakon poroda te odgovorite na temelju svojih osjećaja, iskustava i potreba u tom razdoblju.

☐ Da ☐ Djelomično ☐ Ne

IV. EMOCIONALNA PODRŠKA

Tko Vam je pružao najveću emocionalnu podršku u prvih 14 dana nakon poroda? (moguće više odgovora)

Napomena: Pri odgovaranju na ovo pitanje molimo Vas da se prisjetite razdoblja nakon poroda te odgovorite na temelju svojih osjećaja, iskustava i potreba u tom razdoblju.

☐ Partner ☐ Članovi obitelji

☐ Prijatelji ☐ Primalja

☐ Patronažna sestra ☐ Ostali zdravstveni djelatnici

☐ Nitko

https://doi.org/10.3390/healthcare14213680

U kojoj mjeri ste se osjećali emocionalno podržano u ranom postpartalnom razdoblju? (1 – uopće ne, 5 – u potpunosti)

Napomena: Pri odgovaranju na ovo pitanje molimo Vas da se prisjetite razdoblja nakon poroda te odgovorite na temelju svojih osjećaja, iskustava i potreba u tom razdoblju.

☐ 1 ☐ 2 ☐ 3 ☐ 4 ☐ 5

Jeste li imali osobu kojoj ste se mogli povjeriti o svojim osjećajima nakon poroda?

Napomena: Pri odgovaranju na ovo pitanje molimo Vas da se prisjetite razdoblja nakon poroda te odgovorite na temelju svojih osjećaja, iskustava i potreba u tom razdoblju.

☐ Da ☐ Ne ☐ Ponekad

U kojoj mjeri je socijalna podrška smanjila Vaš osjećaj stresa ili emocionalne nestabilnosti? (1 – nimalo, 5 – u potpunosti)

Napomena: Pri odgovaranju na ovo pitanje molimo Vas da se prisjetite razdoblja nakon poroda te odgovorite na temelju svojih osjećaja, iskustava i potreba u tom razdoblju.

☐ 1 ☐ 2 ☐ 3 ☐ 4 ☐ 5

Smatrate li da je nedostatak socijalne podrške utjecao na pojavu ili intenzitet Vaših emocionalnih poteškoća?

Napomena: Pri odgovaranju na ovo pitanje molimo Vas da se prisjetite razdoblja nakon poroda te odgovorite na temelju svojih osjećaja, iskustava i potreba u tom razdoblju.

☐ Da, značajno ☐ Da, djelomično ☐ Ne ☐ Nisam sigurna

V. EMOCIONALNO STANJE

Označite u kojoj mjeri ste u prvih 14 dana nakon poroda doživljavali sljedeće osjećaje: (1 – uopće ne, 5 – vrlo izraženo)

Učestali plač bez jasnog razloga.

Napomena: Pri odgovaranju na ovo pitanje molimo Vas da se prisjetite razdoblja nakon poroda te odgovorite na temelju svojih osjećaja, iskustava i potreba u tom razdoblju.

☐ 1 ☐ 2 ☐ 3 ☐ 4 ☐ 5

Promjene raspoloženja.

Napomena: Pri odgovaranju na ovo pitanje molimo Vas da se prisjetite razdoblja nakon poroda te odgovorite na temelju svojih osjećaja, iskustava i potreba u tom razdoblju.

☐ 1 ☐ 2 ☐ 3 ☐ 4 ☐ 5

Osjećaj tuge ili praznine.

Napomena: Pri odgovaranju na ovo pitanje molimo Vas da se prisjetite razdoblja nakon poroda te odgovorite na temelju svojih osjećaja, iskustava i potreba u tom razdoblju.

☐ 1 ☐ 2 ☐ 3 ☐ 4 ☐ 5

Osjećaj tjeskobe ili unutarnjeg nemira.

Napomena: Pri odgovaranju na ovo pitanje molimo Vas da se prisjetite razdoblja nakon poroda te odgovorite na temelju svojih osjećaja, iskustava i potreba u tom razdoblju.

☐ 1 ☐ 2 ☐ 3 ☐ 4 ☐ 5

Osjećaj nesigurnosti u vlastitu sposobnost brige za dijete.

Napomena: Pri odgovaranju na ovo pitanje molimo Vas da se prisjetite razdoblja nakon poroda te odgovorite na temelju svojih osjećaja, iskustava i potreba u tom razdoblju.

https://doi.org/10.3390/healthcare14213680

☐ 1 ☐ 2 ☐ 3 ☐ 4 ☐ 5

Koliko su navedeni osjećaji utjecali na Vaše svakodnevno funkcioniranje?

Napomena: Pri odgovaranju na ovo pitanje molimo Vas da se prisjetite razdoblja nakon poroda te odgovorite na temelju svojih osjećaja, iskustava i potreba u tom razdoblju.

☐ Nisam imala navedene osjećaje ☐ Nimalo ☐ Blago ☐ Umjereno ☐ Značajno

Koliko dugo su navedeni osjećaji trajali?

Napomena: Pri odgovaranju na ovo pitanje molimo Vas da se prisjetite razdoblja nakon poroda te odgovorite na temelju svojih osjećaja, iskustava i potreba u tom razdoblju.

☐ Nisam imala navedene osjećaje
☐ Manje od 3 dana
☐ 3–7 dana
☐ 8–14 dana
☐ Dulje od 14 dana

VI. PERCEPCIJA POVEZANOSTI DOJENJA I EMOCIONALNOG STANJA

Smatrate li da su poteškoće s dojenjem utjecale na Vaše emocionalno stanje?

Napomena: Pri odgovaranju na ovo pitanje molimo Vas da se prisjetite razdoblja nakon poroda te odgovorite na temelju svojih osjećaja, iskustava i potreba u tom razdoblju.

☐ Da, značajno
☐ Da, djelomično
☐ Ne
☐ Nisam imala poteškoće s dojenjem

Osjećaj krivnje zbog poteškoća s dojenjem:

Napomena: Pri odgovaranju na ovo pitanje molimo Vas da se prisjetite razdoblja nakon poroda te odgovorite na temelju svojih osjećaja, iskustava i potreba u tom razdoblju.

☐ Uopće ne ☐ Blago ☐ Umjereno ☐ Izrazito

Smatrate li da bi bolja podrška u dojenju smanjila Vaše emocionalne poteškoće?

Napomena: Pri odgovaranju na ovo pitanje molimo Vas da se prisjetite razdoblja nakon poroda te odgovorite na temelju svojih osjećaja, iskustava i potreba u tom razdoblju.

☐ Da ☐ Ne ☐ Nisam sigurna

VII. ZAVRŠNO PITANJE (OPCIONALNO)

Imate li potrebu dodatno opisati svoje iskustvo dojenja i emocionalnog stanja nakon poroda?

Napomena: Pri odgovaranju na ovo pitanje molimo Vas da se prisjetite razdoblja nakon poroda te odgovorite na temelju svojih osjećaja, iskustava i potreba u tom razdoblju.

Zahvaljujemo na sudjelovanju u istraživanju. Vaš doprinos pomaže unapređenju skrbi za rodilje i novorođenčad u Republici Hrvatskoj.

https://doi.org/10.3390/healthcare14213680

PART B | ENGLISH TRANSLATION

BREASTFEEDING AND EMOTIONAL DIFFICULTIES

Dear participant,

This survey is part of a study on the association between breastfeeding difficulties and emotional difficulties in the early postpartum period in the Republic of Croatia. Participation in this study is entirely voluntary and anonymous, and the collected data will be used for scientific purposes only. There are no right or wrong answers in this questionnaire. Please try to recall your experiences as accurately as possible the period after childbirth and your feelings, experiences and needs during that period.

Women aged 18 years or older who have given birth within the preceding 12 months are eligible to participate in this study. Completing the survey takes approximately 7–10 minutes. If you wish to participate in this study, please tick the electronic consent to participate and continue with the questionnaire. You may withdraw from the study at any time.

We thank you for your time, honesty and participation in this study.

I. GENERAL INFORMATION

Age (in years): ___

Level of education:

☐ Primary school ☐ Secondary school
☐ College / undergraduate degree ☐ Master's degree
☐ Postgraduate degree

Employment status:

☐ Employed ☐ Unemployed
☐ Student ☐ Maternity/parental leave

Place of residence:

☐ City ☐ Suburban area ☐ Rural area

II. INFORMATION ON CHILDBIRTH AND THE NEWBORN

Is this your first childbirth? ☐ Yes ☐ No

If you have an older child, did you breastfeed that child? ☐ Yes ☐ No

Mode of birth:

☐ Vaginal birth ☐ Caesarean section
☐ Vaginal birth with episiotomy

Gestational week in which the birth was completed: ___

Was the infant placed with you after birth (rooming-in)? ☐ Yes ☐ No

III. BREASTFEEDING

Did you plan to breastfeed during pregnancy? ☐ Yes ☐ No ☐ I had not considered it

Infant feeding method during the first 7 days after birth:

☐ Exclusive breastfeeding ☐ Breastfeeding with formula supplementation ☐ Formula only

Did you experience breastfeeding difficulties during the first 14 days after birth?

Note: When answering this question, please recall the period after childbirth and answer on the basis of your feelings, experiences and needs during that period.

☐ Yes ☐ No

If YES, please indicate which ones (more than one answer possible):

Note: When answering this question, please recall the period after childbirth and answer on the basis of your feelings, experiences and needs during that period.

☐ Sore or damaged nipples ☐ Difficulty with infant latch
☐ Perceived insufficient milk supply ☐ Milk stasis / mastitis
☐ Pain during breastfeeding ☐ Other: ___________

How emotionally burdensome were the breastfeeding difficulties for you? (1 – not at all, 5 – extremely)

Note: When answering this question, please recall the period after childbirth and answer on the basis of your feelings, experiences and needs during that period.

☐ 1 ☐ 2 ☐ 3 ☐ 4 ☐ 5

Did you receive professional breastfeeding support?

Note: When answering this question, please recall the period after childbirth and answer on the basis of your feelings, experiences and needs during that period.

☐ Yes, a midwife ☐ Yes, a community health nurse
☐ Yes, a breastfeeding counsellor ☐ No

Do you consider that you received sufficient breastfeeding support?

Note: When answering this question, please recall the period after childbirth and answer on the basis of your feelings, experiences and needs during that period.

☐ Yes ☐ Partly ☐ No

IV. EMOTIONAL SUPPORT

Who provided you with the greatest emotional support during the first 14 days after birth? (more than one answer possible)

Note: When answering this question, please recall the period after childbirth and answer on the basis of your feelings, experiences and needs during that period.

☐ Partner ☐ Family members
☐ Friends ☐ Midwife
☐ Community health nurse ☐ Other health professionals
☐ No one

To what extent did you feel emotionally supported in the early postpartum period? (1 – not at all, 5 – completely)

Note: When answering this question, please recall the period after childbirth and answer on the basis of your feelings, experiences and needs during that period.

☐ 1 ☐ 2 ☐ 3 ☐ 4 ☐ 5

https://doi.org/10.3390/healthcare14213680

Did you have a person with whom you could confide about your feelings after childbirth?

Note: When answering this question, please recall the period after childbirth and answer on the basis of your feelings, experiences and needs during that period.

☐ Yes ☐ No ☐ Sometimes

To what extent did social support reduce your feeling of stress or emotional instability? (1 – not at all, 5 – completely)

Note: When answering this question, please recall the period after childbirth and answer on the basis of your feelings, experiences and needs during that period.

☐ 1 ☐ 2 ☐ 3 ☐ 4 ☐ 5

Do you consider that the lack of social support influenced the occurrence or intensity of your emotional difficulties?

Note: When answering this question, please recall the period after childbirth and answer on the basis of your feelings, experiences and needs during that period.

☐ Yes, significantly ☐ Yes, partly

☐ No ☐ I am not sure

V. EMOTIONAL STATE

Indicate to what extent you experienced the following feelings during the first 14 days after birth: (1 – not at all, 5 – very pronounced)

Frequent crying without a clear reason.

Note: When answering this question, please recall the period after childbirth and answer on the basis of your feelings, experiences and needs during that period.

☐ 1 ☐ 2 ☐ 3 ☐ 4 ☐ 5

Mood changes.

Note: When answering this question, please recall the period after childbirth and answer on the basis of your feelings, experiences and needs during that period.

☐ 1 ☐ 2 ☐ 3 ☐ 4 ☐ 5

Feelings of sadness or emptiness.

Note: When answering this question, please recall the period after childbirth and answer on the basis of your feelings, experiences and needs during that period.

☐ 1 ☐ 2 ☐ 3 ☐ 4 ☐ 5

Feelings of anxiety or inner unrest.

Note: When answering this question, please recall the period after childbirth and answer on the basis of your feelings, experiences and needs during that period.

☐ 1 ☐ 2 ☐ 3 ☐ 4 ☐ 5

Feelings of insecurity about your own ability to care for your baby.

Note: When answering this question, please recall the period after childbirth and answer on the basis of your feelings, experiences and needs during that period.

☐ 1 ☐ 2 ☐ 3 ☐ 4 ☐ 5

https://doi.org/10.3390/healthcare14213680

To what extent did the listed feelings affect your daily functioning?

Note: When answering this question, please recall the period after childbirth and answer on the basis of your feelings, experiences and needs during that period.

☐ I did not experience any of the listed feelings ☐ Not at all ☐ Mildly ☐ Moderately ☐ Significantly

How long did the listed feelings last?

Note: When answering this question, please recall the period after childbirth and answer on the basis of your feelings, experiences and needs during that period.

☐ I did not experience any of the listed feelings
☐ Less than 3 days
☐ 3–7 days
☐ 8–14 days
☐ Longer than 14 days

VI. PERCEPTION OF THE RELATIONSHIP BETWEEN BREASTFEEDING AND EMOTIONAL STATE

Do you consider that breastfeeding difficulties affected your emotional state?

Note: When answering this question, please recall the period after childbirth and answer on the basis of your feelings, experiences and needs during that period.

☐ Yes, significantly
☐ No
☐ Yes, partly
☐ I had no breastfeeding difficulties

Feelings of guilt because of breastfeeding difficulties:

Note: When answering this question, please recall the period after childbirth and answer on the basis of your feelings, experiences and needs during that period.

☐ Not at all ☐ Mildly ☐ Moderately ☐ Extremely

Do you consider that better breastfeeding support would have reduced your emotional difficulties?

Note: When answering this question, please recall the period after childbirth and answer on the basis of your feelings, experiences and needs during that period.

☐ Yes ☐ No ☐ I am not sure

VII. FINAL QUESTION (OPTIONAL)

Would you like to describe your experience of breastfeeding and emotional state after childbirth in more detail?

Note: When answering this question, please recall the period after childbirth and answer on the basis of your feelings, experiences and needs during that period.

Thank you for participating in the study. Your contribution helps improve care for mothers and newborns in the Republic of Croatia.

https://doi.org/10.3390/healthcare14213680
